# Association between diet history and symptoms of individuals having recovered from COVID-19

**DOI:** 10.1186/s41043-023-00365-7

**Published:** 2023-03-24

**Authors:** Ola T. Sahloul, Talaat M. Sahloul

**Affiliations:** Faculty of Specific Education, Damietta University, Specific Education Street, The Second Neighborhood, New Damietta, Egypt

**Keywords:** Coronavirus, Injury symptoms, Food history

## Abstract

**Background:**

Many studies show that people who eat a balanced diet have stronger immunity. The present work aimed to identify the effects of the diet history of COVID-19 patients having recovered from the disease on the occurrence and severity of symptoms.

**Methods:**

The study sample consisted of 346 individuals aged 20–65 years. The participants’ data and answers to an electronic questionnaire regarding their diet history and symptoms were collected. The study focused on four hard symptoms (fever, body pain, cough, and dyspnoea) to investigate the relationship between these symptoms and the consumption of specific immunity foods**.**

**Results:**

Symptoms were reported by 88.1% of the participants eating none of the foods investigated, whereas 85.54% and 85.55% of the individuals with little or intensive food intake, respectively, experienced symptoms.

**Conclusions:**

Intake of specific functional foods might slightly reduce the occurrence of some symptoms.

## Introduction

The coronavirus disease 2019 or COVID-19 is characterized by serious acute respiratory symptoms [1]. This disease originated at the end of 2019 in Wuhan, China [2, 3]. In March 2020, the World Health Organization (WHO) considered the COVID-19 outbreak a global pandemic [4]. Since 14 February 2020, when the first cases of COVID-19 infections were reported in Egypt, the Egyptian people have lived in a different reality [5, 6]. The first symptom usually experienced by COVID-19 patients is pain, particularly headaches, myalgia, or arthralgia [7, 8]. The pain appears 1.6 days after the onset of illness. The second set of symptoms is fever, followed by cough and diarrhea. Then, patients suffer from anosmia, which occurs days after the onset of infection. All symptoms persist for 10 ± 4.9 (mean ± standard deviation) days (range of 3–27 days), except for the fever, cough, and anosmia that last 5.5 ± 4.4 (range of 1–19 days), 7.7 ± 4.3 (range of 1–18 days), and 7.3 ± 5 (range of 1–19 days), respectively [9].

In addition to the outbreak, governments have imposed "lock-downs" to mitigate its virus's spread [10, 11]. Therefore, the prevalence of psychological disorders' symptoms has increased widely (e.g., depression, somatic symptoms, anxiety, negative feelings, emotional exhaustion, and panic disorder) [12–14]. Consequently, these symptoms could potentially disrupt sleep patterns [11, 15]. Plus, a reduction in daily levels of physical activity could also negatively affect sleep [16] and thus contribute to an impaired immune system [17].

The nutritional status, which results from the diet history, can significantly impact the overall health and reduce the risk of developing infections [18]. Healthy nutritional habits help prevent non-communicable diseases, which are risk factors for developing COVID-19 [19]. Additionally, nutrition has been linked to systemic infectious diseases through its effects on the immune system [20]. Thus, malnutrition increases the host’s susceptibility to infectious diseases. These infections negatively affect the metabolism, worsening the nutritional state [21]. Moreover, getting used to a healthy lifestyle is important for reducing cholesterol levels and increasing antioxidant levels from fruits, vegetables, and monounsaturated fatty acids present in fish, nuts, and olive oil [22]. Moreover, the frequent consumption of healthy foods such as vegetables, fruits, and fish contributes to supplying the body with sufficient amounts of essential nutrients and antioxidants [23, 24]. In Damietta (Egypt), most COVID-19 patients have poor nutritional habits and present severe symptoms of fatigue. Additionally, most COVID-19 patients are overweight or obese, and these patients have more severe symptoms of fatigue [25]. Interestingly, the Mediterranean diet is one of the healthiest diets in the world and is associated with lower rates of mortality, obesity, type 2 diabetes mellitus, low-grade inflammation, cancer, Alzheimer’s disease, depression, and COVID-19 [22, 26].

Therefore, the current study aims to identify the effects of the nutritional history of patients having recovered from COVID-19 on the occurrence and intensity of some of their symptoms.

## Subjects and methods

### Study design and participants

This study took place in Egypt (Damietta Governorate). A random sample of 346 individuals who had recovered from COVID-19 was selected. The sample consisted of 212 women and 134 men aged 20 to 65 years, 131 from rural areas, and 215 from urban areas. The illness lasted 14 to 21 days for most people (52.3% subjects).

All participants consented to share their data by sending back the electronic questionnaire. Several specialized faculty members from the Faculty of Specific Education at Damietta University verified the test’s validity. The two tests’ stability coefficient was calculated before using the data. The average time spent filling out the survey was 10 min.

### The study collected

- (1) Personal information (gender, location, age, and illness duration).
- (2) Answers to a structured questionnaire on diet history.

Then, the relationship between the diet history, particularly consuming certain foods, and the degree of some symptoms was determined.

An electronic questionnaire (in the Arabic language) was built using the Google Form application [27, 28] and can be viewed at the following URL: https://docs.google.com/forms/d/e/1FAIpQLSfNudaJ3Hpn1XonEFU-rJkq5zZnywYhDzoV9Y28rQ9NkmQ8LA/viewform?usp=sf_link.

Translated version of the survey.https://docs.google.com/forms/d/e/1FAIpQLSd9gyg9-FqB1fpTzTeXqv6DCwJqROjj6kuAAbZCnjhvWxip1Q/viewform?usp=sf_link.

### COVID-19 cases

COVID-19 cases have been defined as symptomatic (with fever, cough, nasal congestion and runny nose, sore throat, dyspnoea, loss of smell or taste, body pain, and diarrhea) or asymptomatic (defined as a positive PCR or antibody test without typical COVID-19 symptoms) [29].

### Severity and duration of COVID-19 illness

Participants rated their COVID-19 symptoms from three options: asymptomatic, moderate symptoms, and severe symptoms. In addition, they had to indicate the number of days spent presenting COVID-19 symptoms [29].

### Statistical analysis

**Fig. 1:**
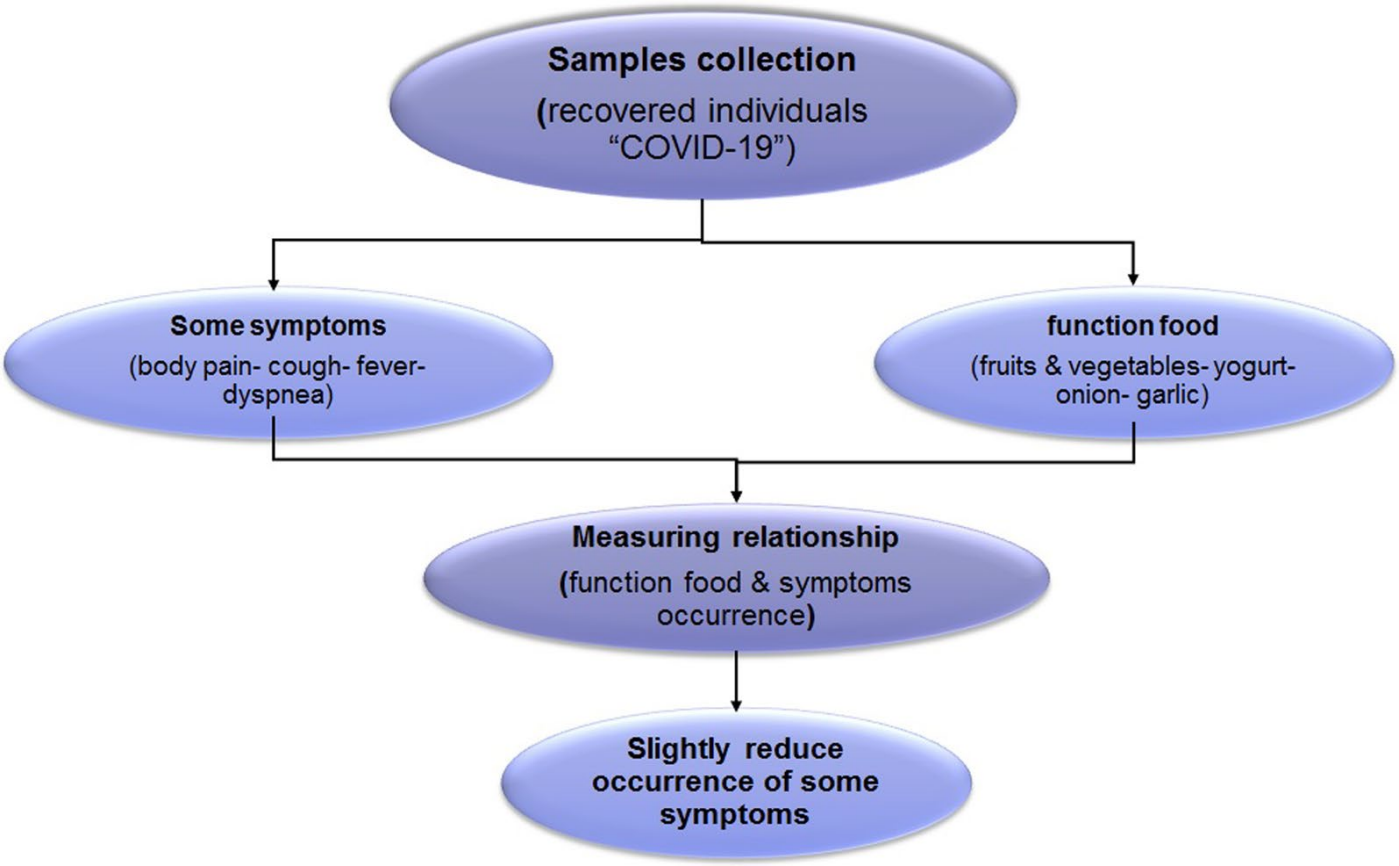
Flowchart to disclosing the relationship between some functional food and COVID-19 symptoms

SPSS statistical software (version 11.5.1) was used to analyse the data collected using Pearson’s correlation coefficient (R) [30] (Fig. 1).

## Results

**Fig. 2:**
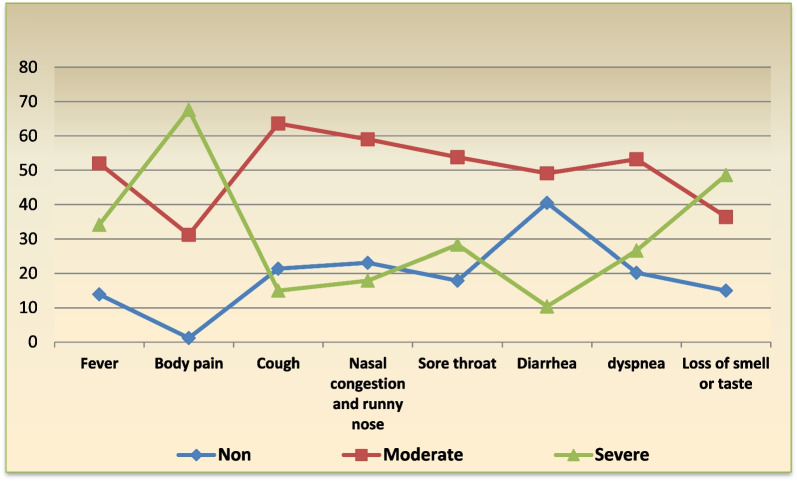
COVID-19 symptoms investigated

Figure 2 recapitulates the COVID-19 symptoms that were investigated, namely fever, body pain, cough, nasal congestion, runny nose, sore throat, diarrhea, dyspnoea, and loss of smell or taste. Most participants reported having had a moderate fever, cough, nasal congestion, runny nose, sore throat, diarrhea, and dyspnoea (52%, 63.6%, 59%, 53.8%, 49.1%, and 53.2%, respectively). Then, the rest of participants had severe body pain and smell or taste loss (67.6% and 48.6%, respectively).

Thus, most participants had moderate symptoms, some had severe symptoms, and a minority was asymptomatic.

**Fig. 3:**
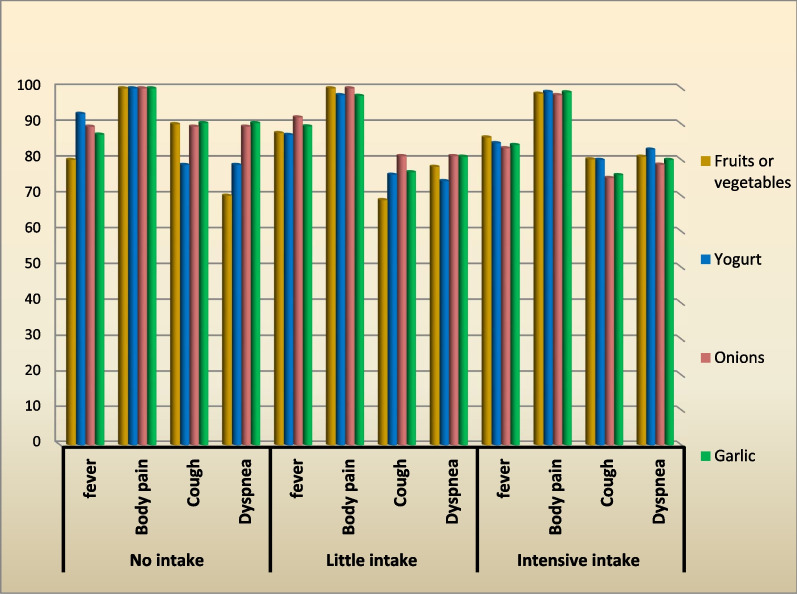
Relationship between the consumption of all investigated foods and the proportion of paticipants having symptoms

Tables 1 to Fig. 3 present the relationship between COVID–19 symptoms (fever, body pain, cough, and dyspnea in Tables 1, 2, 3, and 4, respectively and intake of some foods (fruits or vegetables, yogurt, onions, and garlic). Each table was divided into three levels of food intake: (1) no intake, (2) little intake (weekly or monthly), and (3) intensive intake (more than once a week to daily).

**Table 1:** Relationship between the consumption of specific foods and the occurrence of fever

Level of intake	Foods		Fruits or vegetables		Yogurt		Onions		Garlic		Total symptoms	
	Symptom rate		*N*	%	*N*	%	*N*	%	*N*	%	*N*	%
No intake	Asymptomatic		4	20	2	7.1	6	10.7	8	12.9	20	12
	Symptoms	Moderate	8	40	14	50	26	46.4	32	51.6	80	48.2
		Severe	8	40	12	42.9	24	42.9	22	35.5	66	39.8
		Total	16	80	26	92.9	50	89.3	54	87.1	146	88
		Total	20	100	28	100	56	100	62	100	166	100
Little intake (monthly or weekly)	Asymptomatic		8	12.5	14	13	6	8.1	10	10.6	38	11.2
	Symptoms	Moderate	34	53.1	58	53.7	38	51.4	48	51.1	178	52.3
		Severe	22	34.4	36	33.3	30	40.5	36	38.3	124	36.5
		Total	56	87.5	94	87	68	91.9	84	89.4	302	88.8
		Total	64	100	108	100	74	100	94	100	340	100
Intensive intake (daily or more than once a week)	Asymptomatic	36	13.7	32	15.3	36	16.7	30	15.8	134	15.3	
	Symptoms	Moderate	138	52.7	108	51.4	116	53.7	100	52.6	462	52.6
		Severe	88	33.6	70	33.3	64	29.6	60	31.6	282	32.1
		Total	226	86.3	178	84.7	180	83.3	160	84.2	744	84.7
	Total	262	100	210	100	216	100	190	100	878	100	
Total			346		346		346		346		1384	
*R*			0.063		0.075		0.141**		− 0.068		0.135**	

*N*: Number of participants

*R*: Pearson’s correlation coefficient

^**^^:^* P*-value of correlation was significant *(p* < 0.01), 2-tailed test

**Table 2:** Relationship between the consumption of specific foods and the occurrence of body pain

Level of intake	Foods		Fruits or vegetables		Yogurt		Onions		Garlic		Total symptoms	
	Symptom rate		*N*	%	*N*	%	*N*	%	*N*	%	*N*	%
No intake	Asymptomatic	0	0	0	0	0	0	0	0	0	0	
	Symptoms	Moderate	8	40	8	28.6	14	25	18	29	48	28.9
		Severe	12	60	20	71.4	42	75	44	71	118	71.1
		Total	20	100	28	100	56	100	62	100	166	100
	Total	20	100	28	100	56	100	62	100	166	100	
Little intake (monthly or weekly)	Asymptomatic	0	0	2	1.9	0	0	2	2.1	4	1.2	
	Symptoms	Moderate	16	25	46	42.6	32	43.2	32	34.1	126	37
		Severe	48	75	60	55.5	42	56.8	60	63.8	210	61.8
		Total	64	100	106	98.1	74	100	92	97.9	336	98.8
	Total		64	100	108	100	74	100	94	100	340	100
Intensive intake (daily or more than time weekly)	Asymptomatic	4	1.5	2	1	4	1.9	2	1.1	12	1.4	
	Symptoms	Moderate	84	32.1	54	25.7	62	28.7	58	30.5	258	29.4
		Severe	174	66.4	154	73.3	150	69.4	130	68.4	608	69.2
		Total	258	98.5	208	99	212	98.1	188	98.9	866	98.6
	Total		262	100	210	100	216	100	190	100	878	100
Total			346		346		346		346		1384	
*R*			− 0.057		0.024		0.069		− 0.018		0.037	

*N* Number of participants

*R* Pearson’s correlation coefficient

**Table 3:** Relationship between the consumption of specific foods and the occurrence of cough

Level of intake	Foods		Fruits or vegetables		Yogurt		Onions		Garlic		Total symptoms	
	Symptom rate		*N*	%	*N*	%	*N*	%	*N*	%	*N*	%
No intake	Asymptomatic		2	10	6	21.4	6	10.7	6	9.7	20	12.1
	Symptoms	Moderate	15	75	18	64.3	34	60.7	34	54.8	101	60.8
		Severe	3	15	4	14.3	16	28.6	22	35.5	45	27.1
		Total	18	90	22	78.6	50	89.3	56	90.3	146	87.9
	Total		20	100	28	100	56	100	62	100	166	100
Little intake (monthly or weekly)	Asymptomatic		20	31.2	26	24.1	14	18.9	22	23.4	82	24.1
	Symptoms	Moderate	38	59.4	62	57.4	34	45.9	52	55.3	186	54.7
		Severe	6	9.4	20	18.5	26	35.2	20	21.3	72	21.2
		Total	44	68.8	82	75.9	60	81.1	72	76.6	258	75.9
	Total		64	100	108	100	74	100	94	100	340	100
Intensive intake (daily or more than time weekly)	Asymptomatic		52	19.8	42	20	54	25	46	24.2	194	22.1
	Symptoms	Moderate	167	63.7	98	46.7	110	50.9	92	48.4	467	53.2
		Severe	43	16.4	70	33.3	52	24.1	52	27.4	217	24.7
		Total	210	80.2	168	80	162	75	144	75.8	684	77.9
	Total		262	100	210	100	216	100	190	100	878	100
Total			346		346		346		346		1384	
R			0.022		− 0.086		0.014		− 0.116*		− 0.002	

*N* Number of participants

*R* Pearson’s correlation coefficient

^*^^:^ *P*-value of correlation was significant (*p* < 0.05), 2-tailed test

**Table 4:** Relationship between the consumption of specific foods and the occurrence of dyspnoea

Level of intake	Foods		Fruits or vegetables		Yogurt		Onions		Garlic		Total symptoms	
	Symptoms rate		*N*	%	*N*	%	*N*	%	*N*	%	*N*	%
No intake	Asymptomatic		6	30	6	21.4	10	17.8	14	22.6	36	21.7
	Symptoms	Moderate	8	40	18	64.3	26	46.5	34	54.8	86	51.8
		Severe	6	30	4	14.3	20	35.7	14	22.6	44	26.5
		Total	14	70	22	78.6	46	82.2	48	77.4	130	78.3
	Total	20	100	28	100	56	100	62	100	166	100	
Little intake (monthly or weekly)	Asymptomatic		14	21.9	28	25.9	14	18.9	18	19.1	74	21.8
	Symptoms	Moderate	28	43.8	52	48.2	36	48.6	48	51.1	164	48.2
		Severe	22	34.3	28	25.9	24	32.5	28	29.8	102	30
		Total	50	78.1	80	74.1	60	81.1	76	80.9	266	78.2
	Total	64	100	108	100	74	100	94	100	340	100	
Intensive intake (daily or more than time weekly)	Asymptomatic		50	19.1	36	17.1	46	21.3	38	20	170	19.4
	Symptoms	Moderate	148	56.5	114	54.3	122	56.5	102	53.7	486	55.4
		Severe	64	24.4	60	28.6	48	22.2	50	26.3	222	25.2
		Total	212	80.9	174	82.9	170	78.7	152	80	708	80.6
	Total		262	100	210	100	216	100	190	100	878	100
Total			346		346		346		346		1384	
*R*			0.063		− 0.083		0.091		0.018		0.062*	

*N* Number of participants

*R* Pearson’s correlation coefficient

^*^^:^ *P*-value of correlation was significant (*p* < 0.05), 2-tailed test

Table 1 shows that participants experienced fever independently of their food intake. Data shows that among 166 recovered individuals who did not eat any of the investigated foods, 20 (12.5%) had been asymptomatic, whereas 146 (87.5%) had suffered from either moderate or severe fever. Among the 338 participants with little food intake (weekly or monthly), only 38 cases (11.24%) had no fever, whereas 300 (88.76%) had experienced moderate or severe fever. Finally, among the 877 subjects with intensive food intake (daily or more than once a week), 134 (15.26%) had had no fever, and 744 (84.74%) had suffered from moderate or severe fever .

These data indicate comparable fever occurrence rates (88% and 88.8%) in participants with no or little food uptake, whereas fever occurred less in subjects with intensive intake (84.7%). Additionally, the foods investigated here had different effects on the occurrence of fever. For example, fever was reported less (80%) by participants consuming no vegetables or fruits (no intake), whereas it occurred the most (92.9%) in subjects who did not eat yogurt. Participants with little intake (weekly or monthly) of yogurt, vegetables or fruits (87% and 87.5%, respectively) experienced fever less often. Fevers were more intense in subjects consuming only small amounts of onions and garlic (91.9% and 89.4%, respectively). Finally, 86.3%, 84.7%, 84.2%, and 83.3%) of participants with an intensive intake (daily or more than once a week) of fruits or vegetables, yogurt, garlic, and onion, respectively, had a fever.

There is a significant correlation between fever occurrence, onion, and total food consumption (Pearson’s correlation coefficient *R* = 0.141 and 0.135, respectively, *p* < 0.01).

Data in Table 2 showed that 100% of participants not eating any of the investigated foods (no intake) had moderate or severe body pain symptoms. Moderate or severe body pain was experienced by 98.8% of the participants with little food intake (weekly or monthly) and 98.6% of those with an intensive intake (daily or more than once a week) of the foods. In view of the fact that the proportion of patients suffering from body pain decreased, these foods and their bioactive compounds may have affected the body’s resistance to pain.

Additionally, data in Table 2 reveals that 100% of participants who had experienced body pain were eating few fruits or vegetables and onions (weekly or monthly). In contrast, a slight decrease in the proportion of participants who had suffered from body pain occurred with the intensive consumption of yogurt, garlic, fruits or vegetables, and onions (99%, 98.9%, 98.5%, and 98.1%, respectively).

Data in Table 3 shows Cough symptoms occurred in participants who did or did not eat the investigated foods. According to the findings, 166 recovered individuals who did not consume any food had experienced either a moderate or severe cough. Among the 340 participants having little food intake (weekly or monthly), 258 (75.9%) had suffered from moderate or severe cough symptoms. Finally, among the 878 individuals with intensive food intake (daily or more than once a week), 684 (77.9%) had moderate or severe cough symptoms.

These data revealed a similar cough occurrence in individuals with little or intensive dietary intake (75.9% and 77.9%, respectively). This rate increased in participants eating none of the investigated foods (87.9%). Additionally, the foods tested in this study had different effects on cough occurrence. Participants eating no yogurt reported fewer cough symptoms (78.6%) than those who consumed no garlic, onions, or fruits or vegetables (90.3%, 90%, or 89.3%, respectively). Few individuals appeared to have cough symptoms (68.8%) with little intake (weekly or monthly) of fruits or vegetables, compared to those rarely eating yogurt or garlic (75.9% or 76.6%, respectively). Participants with little onion intake reported experiencing cough symptoms (81.1%). Finally, fewer subjects who often (daily or more than once a week) ate onions or garlic developed cough symptoms (75% and 75.8%, respectively). In contrast, the cough was most experienced by individuals who often had yogurt, fruits or vegetables (80% or 80.2%, respectively). There was a significant correlation between garlic consumption and cough severity (*R* = 0.116, p 0.05).

Data in Table 4 presents the link between the consumption of some foods and the development of dyspnea. Data revealed that 36 out of 166 recovering participants who did not eat the foods did not develop symptoms (21.7%), whereas 130 (78.3%) had suffered from either moderate or severe dyspnea. Among the 340 who had a small intake (weekly or monthly) of foods, only 74 (21.8%) had not had dyspnea, whereas 266 (78.2%) had suffered from either moderate or severe dyspnea. Finally, among the 878 individuals consuming the foods regularly (daily or more than once a week), 170 (19.4%) had not experienced dyspnea, whereas 708 cases (80.6%) developed moderate or severe dyspnea. The data also showed that many individuals had developed dyspnea regardless of their general food consumption (78.3%, 78.2%, and 80.6%, for no, little, and intensive uptake, respectively). Among the participants having no intake of the foods, dyspnea symptoms were less observed in those who did not eat fruits or vegetables (70%), followed by those who did not eat garlic (77.4%), yogurt (78.6%), or onions (82.2%).

Additionally, fewer individuals with a low intake (weekly or monthly) of yogurt reported dyspnea symptoms (74.1%) than those eating little fruit or vegetables, garlic, or onions (78.1%, 80.9%, or 81.1%, respectively). Finally, participants eating regularly (daily or more than once a week) yogurt experienced dyspnea symptoms more often (82.9%) than those eating high levels of onions, garlic, or fruits or vegetables (78.7%, 80.9%, or 80.9%, respectively).

There was a significant correlation between total food consumption and the development of dyspnea (*R* = 0.062, *p* < 0.05).

Figure 3 summarizes the data from Tables 1, 2, 3, and 4 regarding the link between the food consumption history of individuals having recovered from COVID-19 and the rate of specific symptoms. The proportions of individuals who consumed a specific food, never (no intake), sometimes (little intake), or regularly (intensive intake) and developed a specific symptom were calculated. Moreover, the average proportion of participants developing symptoms according to their average intake of specific foods and the average proportion of individuals developing a specific symptom according to their global food consumption level were calculated. The data showed that symptoms were present in 88.1% of participants consuming none of the listed foods, 85.54% of those consuming these foods sometimes, and 85.55% of those eating these foods regularly.

This data draws attention to the fact that the food consumption history of recovered people might have increased their resistance to some symptoms, as fewer participants with little or intensive food intake presented symptoms than those who did not consume any of the foods.

## Discussion

The authors think that this study is the first to link COVID-19 symptoms with consumption levels of some foods by recovered individuals from the disease. Then choose three food intake levels: no intake, little intake (weekly or monthly), and intensive intake (daily or more than once a week) and Four food types that positively affect the immune system were chosen. Indeed, fruits or vegetables provide high amounts of vitamins and minerals that are important for the immune system [31]. Additionally, a lower COVID-19 infection rate in individuals drinking yogurt daily than that of people not drinking yogurt has been reported [32]. Yogurt, a fermented dairy product, exhibits interesting properties related to the presence of bioactive peptides and probiotics that might play a beneficial role in COVID-19 presentation and outcome [33]. Kumar et al*.* (2015) indicated that onions possess immune-stimulatory activities toward murine lymphocytes [34]. Hirayama et al*.* (2019) also suggested that the intake of low or high doses of onion green leaf extract might positively regulate immune competence [35]. Garlic essential oil is also a valuable natural antiviral agent that contributes to preventing the invasion of the human body by a coronavirus [36]. Finally, the immune system is highly affected by malnutrition, which leads to decreased immune responses and a consequent augmented risk of infection and disease severity [37].

On the other hand, participants reported suffering from different symptoms, namely fever, body pain, cough, nasal congestion, runny nose, sore throat, diarrhea, dyspnea, and loss of smell or taste. However, the study found that four symptoms, i.e., fever, body pain, cough, and dyspnea, were more often experienced and were linked with the participants’ history of immune food consumption. These findings were similar to those reported by (Carfì et al*.* 2020) [38], who found that, after the onset of the first COVID-19 symptom, only 18 (12.6%) participants were completely free of any COVID-19 symptom, whereas 32% had 1 or 2 symptoms and 55% had 3 or more. A worse quality of life was observed among 44.1% of the patients. The data also shows that a high proportion of individuals reported myalgia (53.1%), dyspnea (43.4%), and joint pain (27.3%). In this respect, (Çalıca et al*.*, 2020) reported that out of 297 patients, 143 had positive symptoms, and 154 had negative symptoms [39]. The most common symptoms in the positive group were cough (56.6%), weakness (56.6%), taste disorder (35.7%), myalgia (34.3%), and fever (33.6%), whereas in the negative group they were cough (63%), weakness (45.5%), dyspnea (29.9%), headache (27.3%), and fever (24.7%).

The present study compared the occurrence of symptoms according to the general and specific consumption of foods. Although there was no impact of food consumption on symptoms in general, the consumption of specific foods in given amounts affected the occurrence of some symptoms. The most notable difference was body pain, experienced by 100% of the participants who had not eaten any of the foods, whereas it was less present in individuals with low or high food intake. Moreover, as shown in Fig. 3, fewer symptoms were experienced by participants consuming the investigated foods sometimes (little intake) or regularly (intensive intake) compared with the group having no intake of these foods. These data contradict those of a previous study (Kim et al*.*, 2021), indicating that no association was observed between diets and the odds of COVID-19-like illness or duration of symptoms [29].

## Conclusions

The present study suggests that although there is no impact of food consumption on symptoms in general, the consumption of some foods i.e., (Fruits or vegetables, Yogurt, Onions and Garlic) affected the occurrence of some symptoms. The most notable difference is body pain**.**

### Recommendation

The authors suggested to conduct more research by adding more foods related to COVID-19 symptoms especially for children and adolescent. Also, governments should provide more information related immunity foods content in information, education and communication on pandemic targeted to the communities. Furthermore, civil society organizations should pay special attention to assist social including immunity foods vulnerable groups in the family.

### The strengths and limitations of the study are

The strength of the study’s findings lies in the fact that the relationship between different consumption levels of specific foods by people having recovered from COVID-19 and the symptoms experienced by these individuals was investigated.

The limitation of the study was insufficient participants.

## Data Availability

An electronic questionnaire (in the Arabic language) was built using the Google Form application https://docs.google.com/forms/d/e/1FAIpQLSfNudaJ3Hpn1XonEFU-rJkq5zZnywYhDzoV9Y28rQ9NkmQ8LA/viewform?usp=sf_link
